# Pregnancy Vitamin D Supplementation and Childhood Bone Mass at Age 4 Years: Findings From the Maternal Vitamin D Osteoporosis Study (MAVIDOS) Randomized Controlled Trial

**DOI:** 10.1002/jbm4.10651

**Published:** 2022-06-11

**Authors:** Elizabeth M. Curtis, Rebecca J. Moon, Stefania D'Angelo, Sarah R. Crozier, Nicholas J. Bishop, Jaya Sujatha Gopal‐Kothandapani, Stephen H. Kennedy, Aris T. Papageorghiou, Robert Fraser, Saurabh V. Gandhi, Inez Schoenmakers, Ann Prentice, Hazel M. Inskip, Keith M. Godfrey, M. Kassim Javaid, Richard Eastell, Cyrus Cooper, Nicholas C. Harvey, the MAVIDOS Trial Group

**Affiliations:** ^1^ Medical Research Council (MRC) Lifecourse Epidemiology Centre University of Southampton Southampton UK; ^2^ Paediatric Endocrinology University Hospital Southampton National Health Service (NHS) Foundation Trust Southampton UK; ^3^ Academic Unit of Child Health, Sheffield Children's Hospital University of Sheffield Sheffield UK; ^4^ Nuffield Department of Women's & Reproductive Health, John Radcliffe Hospital University of Oxford Oxford UK; ^5^ Department of Obstetrics and Gynaecology, Sheffield Hospitals National Health Service (NHS) Trust University of Sheffield Sheffield UK; ^6^ Department of Medicine, Faculty of Medicine and Health Sciences University of East Anglia Norwich UK; ^7^ Medical Research Council (MRC) Nutrition and Bone Health, Clifford Allbutt Building University of Cambridge Cambridge UK; ^8^ National Institute for Health Research (NIHR) Southampton Biomedical Research Centre University of Southampton and University Hospital Southampton National Health Service (NHS) Foundation Trust Southampton UK; ^9^ Nuffield Department of Orthopaedics, Rheumatology and Musculoskeletal Sciences University of Oxford Oxford UK; ^10^ National Institute for Health Research (NIHR) Oxford Biomedical Research Centre University of Oxford Oxford UK; ^11^ Department of Oncology and Metabolism University of Sheffield Sheffield UK

**Keywords:** CLINICAL TRIALS, DXA, FRACTURE PREVENTION, NUTRITION, OSTEOPOROSIS

## Abstract

In the Maternal Vitamin D Osteoporosis Study (MAVIDOS) randomized trial, vitamin D supplementation in pregnancy did not lead to greater neonatal bone mass across the trial as a whole, but, in a prespecified secondary analysis by season of birth, led to greater neonatal bone mass among winter‐born babies. Demonstrating persistence of this effect into childhood would increase confidence in a long‐term benefit of this intervention. We investigated whether antenatal vitamin D supplementation increases offspring bone mineralization in early childhood in a prespecified, single‐center follow‐up of a double‐blinded, multicenter, randomized controlled clinical trial based in the UK (MAVIDOS). A total of 1123 women in early pregnancy with a baseline 25‐hydroxyvitamin D level 25–100 nmol/L from three research centers (2008–2014) were randomized to 1000 IU/d cholecalciferol or matched placebo from 14 weeks of gestation to delivery. Offspring born at the Southampton, UK research center were assessed at age 4 years (2013–2018). Anthropometry and dual‐energy X‐ray absorptiometry (DXA) were performed (yielding whole body less head [WBLH] bone mineral content [BMC], areal bone mineral density [aBMD], bone area [BA], and body composition). Of 723 children, 564 (78.0%) children attended the 4‐year visit, 452 of whom had a useable DXA. Maternal vitamin D supplementation led to greater WBLH aBMD in the children compared with placebo (mean [95% confidence interval {CI}]: supplemented group: 0.477 (95% CI, 0.472–0.481) g/cm^2^; placebo group: 0.470 (95% CI, 0.466–0.475) g/cm^2^, *p* = 0.048). Associations were consistent for BMC and lean mass, and in age‐ and sex‐adjusted models. Effects were observed across the whole cohort irrespective of season of birth. Maternal‐child interactions were observed, with a greater effect size among children with low milk intake and low levels of physical activity. Child weight, height, and body mass index (BMI) were similar by maternal randomization group. These findings suggest a sustained beneficial effect of maternal vitamin D supplementation in pregnancy on offspring aBMD at age 4 years, but will require replication in other trials. © 2022 The Authors. *JBMR Plus* published by Wiley Periodicals LLC on behalf of American Society for Bone and Mineral Research.

## Introduction

There is increasing evidence that higher maternal vitamin D status during pregnancy leads to improved bone health in the offspring.^(^ ^1^, ^2^ ^)^ Several observational studies, initially in Southampton, UK,^(^ ^3^ ^)^ and subsequently further cohorts in Finland^(^ ^4^, ^5^ ^)^ and Australia,^(^ ^6^ ^)^ have demonstrated associations between maternal 25(OH)‐vitamin D (25(OH)D) status in pregnancy and measures of offspring bone development in childhood. In the Australian Raine cohort, such positive associations were still apparent in young adulthood, at around the age of peak bone mass.^(^ ^6^ ^)^ However, findings across observational studies have not been consistent, notably with null results from Bristol, UK,^(^ ^7^, ^8^ ^)^ and Rotterdam, Netherlands.^(^ ^9^ ^)^ Previous intervention studies have been small and/or inadequately addressed bone outcomes.^(^ ^10^ ^)^ Supported by our comprehensive review of the existing literature,^(^ ^10^ ^)^ we undertook the Maternal Vitamin D Osteoporosis Study (MAVIDOS), a double‐blind, randomized, placebo‐controlled trial of 1000 IU daily vitamin D supplementation in pregnancy in the UK, to test whether maternal vitamin D supplementation in pregnancy would lead to improved offspring bone mass.^(^ ^11^, ^12^ ^)^

In MAVIDOS, although the primary outcome of neonatal whole bone mineral content (BMC) did not differ significantly between babies born to vitamin D supplemented versus placebo mothers, a prespecified secondary analysis^(^ ^12^ ^)^ demonstrated that among winter births, the intervention led to a 0.5 standard deviation (SD) increase in neonatal whole‐body BMC compared with placebo, with no differences apparent in other seasons. Season of birth was one of 10 interactions tested, the others being study center, maternal ethnic origin, parity, treatment compliance, protocol completion, baseline maternal BMI, baseline maternal 25(OH)D, change in 25(OH)D from 14 weeks to 34 weeks, and offspring sex.^(^ ^12^ ^)^ A key question is whether the differences observed at birth persist into later childhood. Sustained differences would increase our confidence in a true biological effect and in the translation for a longer‐term benefit on skeletal health, by improving peak bone mass and thereby reducing future adult fracture risk.^(^ ^1^ ^)^

As planned in the original MAVIDOS trial protocol,^(^ ^11^ ^)^ we followed up children postnatally to investigate whether the maternal pregnancy vitamin D intervention would lead to increased offspring bone mass at 4 years of age. We also investigated any influences on lean and fat mass, and on grip strength, given that these parameters are associated with bone mass.

## Subjects and Methods

### Study design and participants

MAVIDOS is a multicenter, double‐blind, randomized, placebo‐controlled trial of vitamin D supplementation in pregnancy. The primary outcome was neonatal bone mass. A detailed description of the trial protocol^(^ ^11^ ^)^ and primary findings have been published.^(^ ^12^ ^)^ The trial was conducted in accordance with the Declaration of Helsinki guidelines and was approved by the Institutional Review Board (Southampton and South West Hampshire Research Ethics Committee). MAVIDOS was registered prospectively (ISRCTN:82927713; EUDRACT:2007‐001716‐23); full approval from UK Medicines and Healthcare products Regulatory Agency (MHRA) was granted, and all participants gave written, informed consent.^(^ ^12^ ^)^

Women, over 18 years old, attending one of three UK hospitals (University Hospital Southampton NHS Foundation Trust, Oxford University Hospitals NHS Foundation Trust and Sheffield Hospitals NHS Trust) for early pregnancy ultrasound screening (11–14 weeks of gestation) between October 6, 2008 and February 11, 2014 were invited to participate in the study. Inclusion and exclusion criteria have been published.^(^ ^12^, ^13^ ^)^ Participants were randomized in a double‐blind design to either cholecalciferol 1000 IU/d or matched placebo (commenced before 17 weeks of gestation). All participants received standard antenatal care, and could continue self‐administration of dietary supplements containing up to 400 IU/d vitamin D.^(^ ^12^ ^)^

#### Maternal assessments during pregnancy

Detailed maternal phenotyping was performed on the day study medication was dispensed and at 34 weeks of gestation. This including assessment of diet, lifestyle, health and anthropometry, and collection of a non‐fasted blood sample. 25(OH)D was assessed by radioimmunoassay. Full details of the maternal assessments,^(^ ^11^, ^12^ ^)^ assay performance and quality control are given elsewhere.^(^ ^14^, ^15^ ^)^

#### Outcomes at the 4‐year follow‐up visit

As specified in the original trial protocol,^(^ ^11^ ^)^ the children of the Southampton participants were invited to attend the Osteoporosis Centre at Southampton General Hospital for assessment of bone mass and body composition at 4 years of age (March 2013 to October 2018). Parents/guardians remain blinded to their maternal randomization group. Written informed consent was obtained from the parent/guardian. Health, diet, and lifestyle information were collected using an interviewer‐administered questionnaire. Standing height (without shoes) was measured using a portable stadiometer (Leicester height measurer; Seca Ltd, Birmingham, UK), to the nearest 0.1 cm, measured three times and a mean calculated. Weight was measured in light clothing using calibrated electronic scales (Seca Ltd) to the nearest 0.1 kg. Height, weight, and body mass index (BMI) *Z*‐scores for age and sex were calculated using British reference data.^(^ ^16^, ^17^ ^)^ Grip strength was measured three times in each hand, alternating between hands, using a Jamar dynamometer (Promedics, Blackburn, UK).

Whole‐body and lumbar spine dual‐energy X‐ray absorptiometry (DXA) scans were obtained (Hologic Discovery instrument; Hologic Inc., Bedford, MA, USA) in pediatric scan mode. Scans were reviewed by a clinician masked to treatment allocation (EMC/RJM); those with movement artifact were re‐reviewed (NCH). Scans with substantial movement artifact affecting the whole body and/or both legs/both arms were removed from the analysis. In scans with movement artifact in one limb, the region of interest (ROI) of the unaffected limb was transposed into that of the limb with movement artifact.

### Statistical analysis

Baseline characteristics by randomization group were assessed by inspection. Comparisons between attendees and non‐attendees, and of child outcomes by maternal randomization group, were performed using *t* tests, Mann‐Whitney *U* tests, and chi‐square tests for normally distributed continuous, non‐normally distributed continuous and categorical variables, respectively. DXA outcomes and grip strength were transformed to a standard deviation scale for ease of comparison of effect sizes in regression models. DXA measures included whole body less head (WBLH) bone area (BA), BMC, areal bone mineral density (aBMD), and size‐corrected BMC (BMC adjusted for BA, height, and weight [scBMC]), together with total lean mass and fat mass. Both maximum and mean grip strength values were analyzed. In order to increase precision in our estimates of bone outcomes, we included offspring sex and age at DXA in regression models. Grip strength was adjusted for height and sex before inclusion in the models.^(^ ^18^ ^)^

We hypothesized that there might be interactions between maternal randomization group and each of the following: (i) season of delivery (since background 25(OH)D concentration varies by season, and an interaction was observed on neonatal bone measures^(^ ^12^ ^)^); (ii) maternal baseline 25(OH)D (because achieved 25(OH)D is partly dependent on baseline^(^ ^19^ ^)^); (iii) child's calcium intake at 4 years of age (because the effect of maternal vitamin D supplementation on bone metabolism is influenced by calcium intake^(^ ^20^ ^)^); and (iv) child's physical activity at 4 years of age (because an influence of physical activity and interactions between calcium intake and physical activity on bone have been documented^(^ ^21^, ^22^ ^)^). We defined season of birth using the UK Meteorological Office classification, as winter (December–February), spring (March–May), summer (June–August), and autumn (September–November) (https://www.metoffice.gov.uk/), in keeping with our previous analysis of bone mass at birth.^(^ ^12^ ^)^ In order to maximize power in this subset, we also dichotomized the seasons into “winter/spring” (the months in which 25(OH)D concentrations tend to be lowest, December–May) and “summer/autumn” (the months in which 25(OH)D concentrations tend to be highest, June–November), using UK Meteorological office recommendations. Given the effect of body size on DXA measures, we undertook sensitivity analyses controlling for child's height or weight. Analysis of our safety outcomes has been published.^(^ ^12^ ^)^ Stata V15.1 (StataCorp LP, College Station, TX, USA) was used for all analyses.

### Role of the funding source

The study was funded by Versus Arthritis, UK Medical Research Council, UK National Institute for Health Research, with further funding from the Bupa Foundation, UK Biotechnology and Biological Sciences Research Council, and European Union (EU). The original protocol incorporated suggestions from the Arthritis Research UK Clinical Trials Collaboration. The funders had no role in data collection, data analysis, data interpretation, or writing of the report. The corresponding author had full access to all the data in the study and had final responsibility for the decision to submit for publication.

## Results

### Characteristics of the participants

**Fig. 1:**
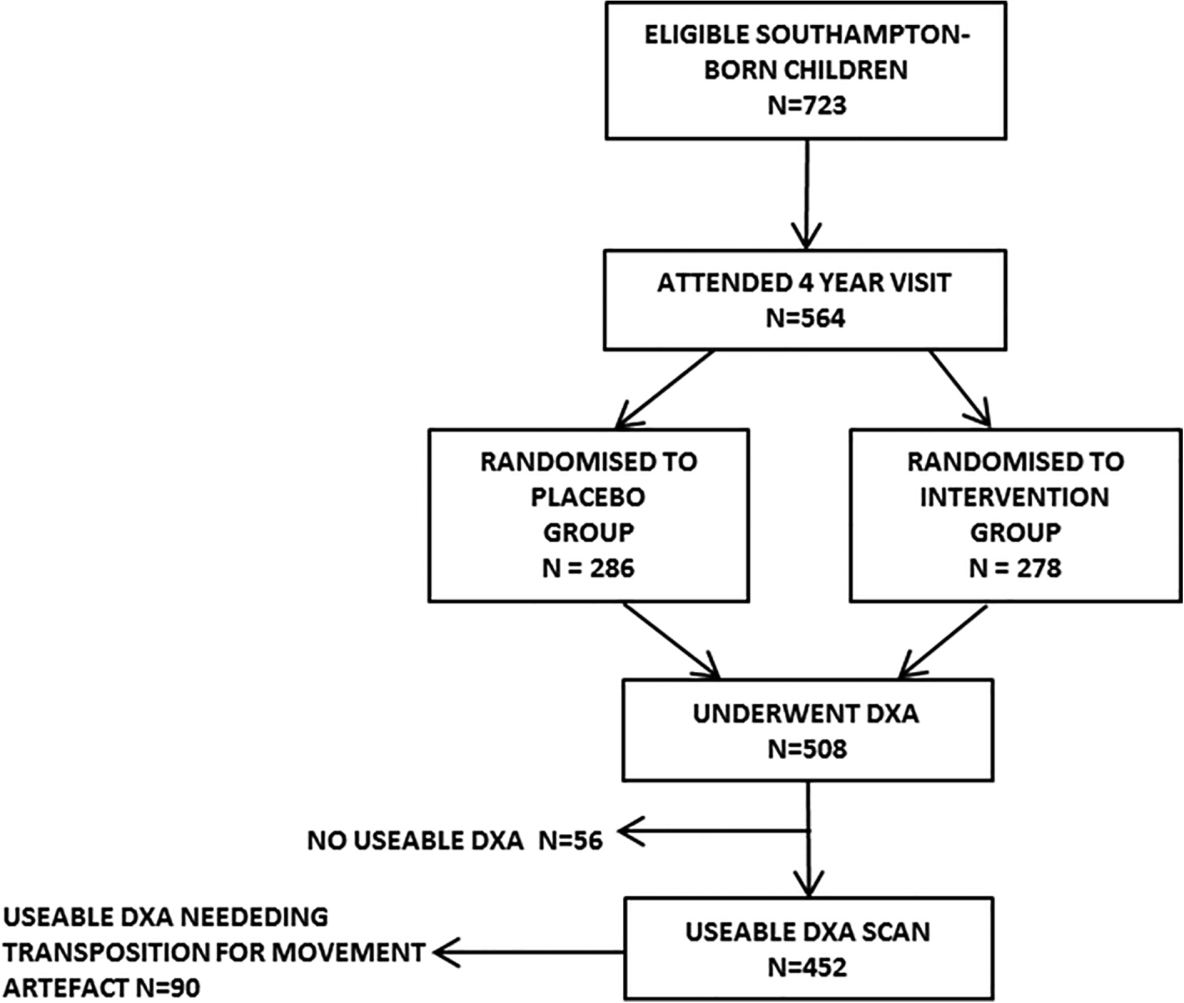
MAVIDOS trial consort diagram for the Southampton‐based 4‐year follow‐up. Detailed flow through the trial including dropout is given in Cooper and colleagues.^(^ ^12^ ^)^

A total of 723 babies were born at term at the Southampton research center; 564 (78.0% of eligible children) attended the 4‐year visit (cholecalciferol group = 278; placebo group = 286). Of these, 508 children (90.1% of attendees) underwent DXA scanning, and 452 children had a useable DXA scan (89.0% of all DXAs). Ninety DXAs (19.9% of the useable DXAs) had movement artifact in one upper/lower limb, so data from the ROI of the opposite side were used, as outlined in Fig. 1. Maternal characteristics were similar between the two randomization groups (Table 1). Table S1 demonstrates the comparison of maternal characteristics between those attending and not attending the 4‐year visit. Mothers attending the 4‐year visit were of older age at delivery, higher educational attainment, and were less likely to smoke in pregnancy compared to non‐attenders. When analyzed by randomization group, mothers attending the 4‐year visit in the placebo group were more likely to be of white ethnicity and hence taller height. Table 2*A* shows the characteristics of the boys and girls attending the 4‐year visit; boys were taller and heavier than girls. When stratified by sex (Table 2*B*,*C*), there were no differences between the placebo and cholecalciferol groups in terms of offspring age, gestational age at birth, weight, height, duration of breastfeeding, and milk consumption at age 4 years. In terms of vitamin D supplementation in childhood, 106 (37.2%) children in the placebo and 102 (37.1%) children in the maternal cholecalciferol supplemented group took a vitamin supplement (of any type) which was balanced between groups, *p* difference = 0.98.

**Table 1:** Characteristics of the Mothers of the Children Attending the MAVIDOS 4‐Year Follow‐Up

Characteristic	*n*	Placebo	*n*	Cholecalciferol 1000 IU/d
Maternal age (years), mean ± SD	286	32.1 ± 4.7	278	32.0 ± 4.7
White ethnicity, *n* (%)	269	260 (96.7)	263	248 (94.3)
Nulliparous, *n* (%)	267	114 (42.7)	265	115 (43.4)
Educated to A level or higher, *n* (%)	266	216 (81.2)	264	221 (83.7)
Height (m), mean ± SD	265	166.3 ± 6.4	266	165.6 ± 6.3
BMI (kg/m^2)^, median (IQR)	265	25.5 (22.8, 29.6)	266	24.9 (22.3, 28.5)
Early pregnancy smoking, *n* (%)	268	14 (5.2)	265	11 (4.2)
Late pregnancy smoking, *n* (%)	254	13 (5.1)	245	12 (4.9)
Moderate/strenuous physical activity in LP (hours/week)	181	0.83 (0.52)	174	0.88 (0.74)
Use of vitamin D supplements, *n* (%)^a^	269	164 (61.0)	266	163 (61.3)
Maternal vitamin D, median (IQR)				
EP 25(OH)D (nmol/L)	280	45.1 (33.9, 56.4)	273	45.0 (33.9, 57.4)
LP 25(OH)D (nmol/L)	257	42.4 (23.3, 56.4)	252	67.4 (56.2, 80.3)

All measures at baseline (EP) unless stated otherwise.

BMI = body mass index; EP = early pregnancy, 14 weeks; IQR = interquartile range; LP = late pregnancy, 34 weeks.

^a^ Personal supplements up to 400 IU/d in addition to study medication.

**Table 2:** Characteristics of the Boys and Girls of the Southampton Arm of the MAVIDOS Trial Attending the 4‐Year Follow‐Up Visit, Demonstrating the Differences in Characteristics between the Sexes (*A*), and by Sex According to Group (*B* and *C*)

(*A*)	*n*	Boys	*n*	Girls	*p* difference
Age (years), median (IQR)	303	4.1 (4.0, 4.2)	257	4.1 (4.0, 1.2)	0.33
Gestational age at birth (weeks), median (IQR)	305	40.4 (39.3, 41.1)	258	40.3 (39.3, 41.0)	0.32
Weight (kg), mean ± SD	302	17.5 ± 2.1	258	17.1 ± 2.2	0.02
Height (cm), mean ± SD	301	105.5 ± 4.3	254	104.3 ± 4.5	0.002
BMI (kg/m^2^), mean ± SD	301	15.7 ± 1.2	254	15.6 ± 1.3	0.76
Duration breastfed (months), median (IQR)	267	5 (1, 11)	230	5 (1, 10)	0.64
Milk consumption at 4 years (pints/d), median (IQR)	305	0.5 (0.35, 0.75)	259	0.5 (0.35, 0.75)	0.91

	Boys				
(*B*)	*n*	Placebo	*n*	Cholecalciferol	*p* difference
Age (years), median (IQR)	142	4.1 (4.0, 4.2)	161	4.1 (4.0, 4.1)	0.21
Gestational age at birth (weeks), median (IQR)	144	40.4 (39.3, 41.1)	161	40.4 (39.3, 41.1)	0.92
Weight (kg), mean ± SD	143	17.4 ± 1.8	159	17.5 ± 2.3	0.74
Height (cm), mean ± SD	143	105.5 ± 4.2	158	105.6 ± 4.3	0.82
BMI (kg/m^2^), mean ± SD	143	15.7 ± 1.1	158	15.7 ± 1.3	0.87
Duration breastfed (months), median (IQR)	120	4 (0.5, 9)	147	6 (1, 12)	0.06
Milk consumption at 4 years (pints/d), median (IQR)	144	0.5 (0.3, 0.8)	161	0.5 (0.4, 0.8)	0.14

	Girls				
(*C*)	*n*	Placebo	*n*	Cholecalciferol	*p* difference
Age (years), median (IQR)	142	4.1 (4.0, 4.2)	115	4.1 (4.0, 4.2)	0.64
Gestational age at birth (weeks), median (IQR)	141	40.3 (39.3, 41)	117	40.3 (39.4, 41)	0.89
Weight (kg), mean ± SD	142	17.0 ± 2.3	116	17.2 ± 2.1	0.45
Height (cm), mean ± SD	138	104.1 ± 4.5	116	104.7 ± 4.4	0.29
BMI (kg/m^2^), mean ± SD	138	15.6 ± 1.4	116	15.6 ± 1.2	0.97
Duration breastfed (months), median (IQR)	124	4 (0, 9.5)	106	6 (1, 10)	0.19
Milk consumption at 4 years (pints/d), median (IQR)	142	0.5 (0.4, 0.8)	117	0.5 (0.4, 0.8)	0.54

BMI = body mass index; IQR = interquartile range.

### Differences in maternal 25(OH)‐vitamin D concentrations across pregnancy

Maternal plasma 25(OH)D concentrations at baseline did not differ by randomization group (median [interquartile range {IQR}]: cholecalciferol group: 45.0 [33.9 to 57.4] nmol/L; placebo group: 45.1 [33.9 to 56.4] nmol/L). 25(OH)D in late pregnancy was higher in the cholecalciferol group (median [IQR]: 67.4 [56.2 to 80.3] nmol/L) compared with placebo (42.4 [23.3 to 56.4] nmol/L), as shown in Table 1.

### Maternal vitamin D supplementation and offspring bone indices, lean mass and grip strength at 4 years of age

**Fig. 2:**
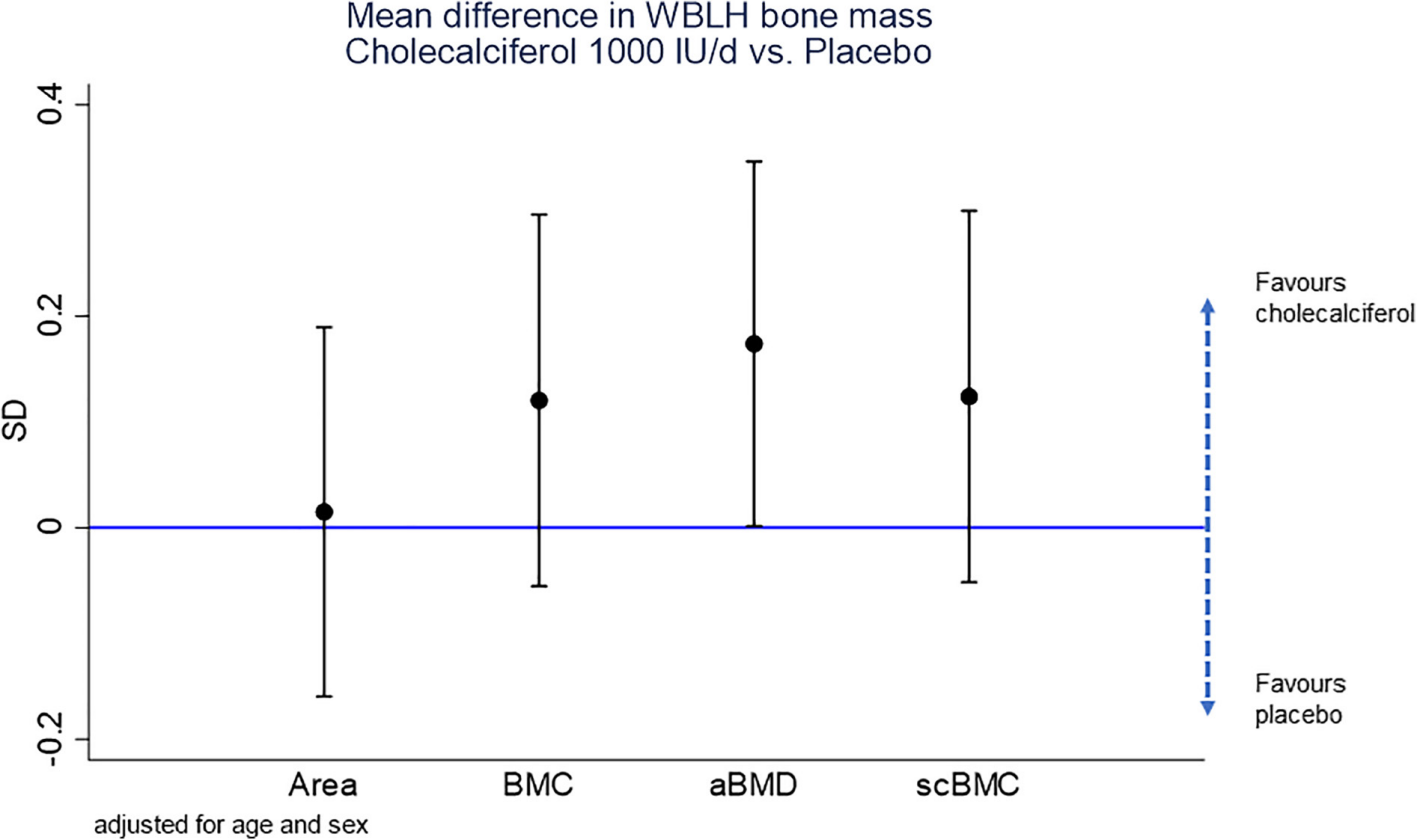
Mean (95% CI) difference (SD) in 4‐year DXA outcomes for cholecalciferol versus placebo group offspring. Each bar is the outcome of a separate linear regression adjusted for age and sex, outcomes are expressed in SDs (SD, 95% CI). Area = bone area; BMC = bone mineral content; BMD = bone mineral density.

Table 3*A* summarizes the crude differences in anthropometry, bone and body composition measures, and grip strength at 4 years of age by maternal randomization group. WBLH aBMD was greater in the offspring of mothers randomized to cholecalciferol in pregnancy compared with placebo (mean [95% CI]: 0.477 [0.472 to 0.481] versus 0.470 [0.466 to 0.475] g/cm^2^, respectively, *p* = 0.05). Because there was a numerically greater percentage (*p* = 0.08) of boys in the cholecalciferol group, Table 3*B* was stratified by sex. Greater BMC, aBMD, and scBMC in the cholecalciferol group compared to the placebo group was observed in both sexes (265 boys with DXA, 229 girls with DXA); however, these differences were not statistically significant. In linear regression models, including all children, adjusting for sex and age at DXA, the positive effect of cholecalciferol supplementation on offspring WBLH aBMD persisted (β: 0.17 [95% CI, 0.002 to 0.35] SD, *p* = 0.05) (Table 4 *A*, Fig. 2). This difference was attenuated by adjustment for the child's height or weight (Table S2). Associations between cholecalciferol supplementation and WBLH BMC (β: 0.12 [95% CI, −0.06 to 0.30] SD, *p* = 0.18) and WBLH scBMC (β: 0.12 [95% CI, −0.06 to 0.30] SD, *p* = 0.17) were in the same positive direction as WBLH aBMD, but were nonstatistically significant.

**Table 3:** Demographic, Anthropometric, Bone, and Body Composition Characteristics of the Children at 4 Years, by Maternal Randomization Group in (*A*) All Children, and (*B*) Stratified by Sex

(*A*)	*n*	Placebo	*n*	Cholecalciferol 1000 IU/d	*p* difference
Age (years), median (IQR)	284	4.1 (4.0, 4.2)	276	4.1 (4.0, 4.2)	0.61
Male sex, %	285	50.5	278	57.9	0.08
Height (cm), mean ± SD	281	104. 8 ± 4.4	274	105.2 ± 4.4	0.27
Height for age/sex *Z*‐score, mean ± SD	279	0.46 ± 1.06	272	0.58 ± 1.06	0.21
Weight (kg), mean ± SD	285	17.2 ± 2.1	275	17.4 ± 2.2	0.34
Weight for age/sex *Z*‐score, mean ± SD	283	0.21 ± 0.92	273	0.28 ± 1.04	0.36
BMI (kg/m^2^), mean ± SD	281	15.6 ± 1.3	274	15.7 ± 1.2	0.91
BMI for age/sex *Z*‐score, mean ± SD	243	0.14 ± 1.15	214	0.10 ± 1.71	0.74
Bone outcomes: whole body (less head), mean ± SD					
BA (cm^2^)	246	756.7 ± 51.7	248	756.0 ± 53.5	0.88
BMC (g)	246	356.7 ± 43.6	248	361.2 ± 44.1	0.25
aBMD (g/cm^2^)	246	0.470 ± 0.037	248	0.477 ± 0.036	0.048
scBMC (g)	243	237.6 ± 17.2	248	239.7 ± 17.9	0.19
Body composition: whole body (less head)					
Lean (g), mean ± SD	248	9006.3 ± 1408.1	248	9248.2 ± 1345.2	0.05
Fat (g), median (IQR)	248	4516.9 (3882.8, 5360.0)	248	4446.9 (3779.8, 5276.2)	0.52
Grip strength, mean ± SD					
Maximum (kg)	262	5.7 ± 1.9	253	5.9 ± 1.9	0.27
Mean (of 6 attempts) (kg)	262	4.5 ± 1.6	253	4.7 ± 1.5	0.33

	Boys					Girls				
(*B*)	*n*	Placebo	*n*	Cholecalciferol 1000 IU/d	*p* difference	*n*	Placebo	*n*	Cholecalciferol 1000 IU/d	*p* difference
Age (years), median (IQR)	142	4.1 (4.0, 4.2)	161	4.1 (4.0, 4.1)	0.21	142	4.1 (4.0, 4.2)	115	4.1 (4.0, 4.2)	0.64
Height (cm), mean ± SD	143	105.4 ± 4.2	158	105.6 ± 4.3	0.82	138	104.1 ± 4.5	116	104.7 ± 4.4	0.29
Height for age/sex *Z*‐score, mean ± SD	141	0.5 ± 1.0	158	0.6 ± 1.0	0.60	138	0.4 ± 1.1	114	0.6 ± 1.1	0.23
Weight (kg), mean ± SD	143	17.4 ± 1.8	159	17.5 ± 2.3	0.73	142	17.0 ± 2.3	116	17.2 ± 2.1	0.45
Weight for age/sex *Z*‐score, mean ± SD	141	0.3 ± 0.8	159	0.3 ± 1.0	0.82	142	0.2 ± 1.0	114	0.3 ± 1.0	0.31
BMI (kg/m^2^), mean ± SD	143	15.7 ± 1.1	158	15.7 ± 1.3	0.87	138	15.6 ± 1.4	116	15.6 ± 1.2	0.97
BMI for age/sex *Z*‐score, mean ± SD	126	0.03 ± 1.3	125	0.08 ± 1.3	0.76	117	0.3 ± 1.0	89	0.1 ± 2.2	0.54
Bone outcomes: whole body (less head), mean ± SD										
BA (cm^2^)	125	749.7 ± 53.5	140	748.2 ± 55.7	0.82	121	764.0 ± 49.0	108	766.1 ± 48.8	0.74
BMC (g)	125	359.1 ± 44.2	140	361.6 ± 46.6	0.66	121	354.2 ± 43.1	108	360.8 ± 40.7	0.24
aBMD (g/cm^2^)	125	0.478 ± 0.033	140	0.482 ± 0.037	0.31	121	0.463 ± 0.039	108	0.470 ± 0.032	0.13
scBMC (g)	123	240.3 ± 17.5	140	241.5 ± 17.1	0.58	120	235.0 ± 16.6	108	237.4 ± 18.7	0.29
Body composition: whole body (less head)										
Lean (g), mean ± SD	125	9544.8 ± 1224.5	140	9657.8 ± 1367.0	0.48	123	8459.0 ± 1375.1	108	8717.3 ± 1115.9	0.12
Fat (g), median (IQR)	123	4238.5 (3662.3, 4917.4)	140	4170.1 (3615.1, 4916.6)	0.86	125	4840.6 (4161.4, 5551.1)	108	4846.4 (4204.3, 5629.1)	0.87
Grip strength, mean ± SD										
Maximum (kg)	131	6.1 ± 1.9	145	6.2 ± 2.0	0.84	131	5.4 ± 1.8	108	5.6 ± 1.6	0.29
Mean (of 6 attempts) (kg)	131	4.9 ± 1.6	145	4.9 ± 1.7	0.86	131	4.2 ± 1.6	108	4.4 ± 1.3	0.21

IQR = interquartile range.

**Table 4:** Associations Between Maternal Treatment Group (Cholecalciferol 1000 IU/d Versus Placebo) and Whole‐Body‐Less‐Head DXA/Body Composition Outcomes in Their Children Assessed at age 4 years (*A*) in All Children and (*B*) Stratified by Sex

	Cholecalciferol versus placebo			
(*A*)	Adjusted for age and sex			
WBLH DXA outcomes	*n*	β (SD)	95% CI	*p*
BA	489	0.01	−0.16, 0.19	0.87
BMC	489	0.12	−0.06, 0.30	0.18
aBMD	489	0.17	0.00, 0.35	0.05
scBMC	486	0.12	−0.05, 0.30	0.17
Lean	491	0.15	−0.02, 0.31	0.08
Fat	491	−0.01	−0.18, 0.16	0.91

	Cholecalciferol versus placebo							
(*B*)	Boys: adjusted for age				Girls: adjusted for age			
WBLH DXA outcomes	*n*	β (SD)	95% CI	*p*	*n*	β (SD)	95% CI	*p*
BA	263	−0.00	−0.25, 0.24	0.97	226	0.04	−0.21, 0.28	0.77
BMC	263	0.07	−0.18, 0.32	0.58	226	0.18	−0.07, 0.43	0.16
aBMD	263	0.13	−0.10, 0.37	0.26	226	0.22	−0.04, 0.48	0.10
scBMC	261	0.09	−0.15, 0.33	0.45	225	0.16	−0.10, 0.42	0.23
Lean	263	0.09	−0.13, 0.31	0.42	228	0.21	−0.03, 0.46	0.09
Fat	261	0.04	−0.20, 0.27	0.76	230	−0.06	−0.31, 0.19	0.64

scBMC = size‐corrected bone mineral content (BMC for bone area, height, and weight).

Lean mass was also greater among the intervention group children (mean [95% CI]: 9248.2 [95% CI, 9080.0 to 9416.5] versus 9006.3 [95% CI, 8830.2 to 9182.4] g, respectively, *p* = 0.05), although attenuated by adjustment for age and sex (β = 0.15 [95% CI, −0.02 to 0.31] SD, *p* = 0.08; and further attenuated by adjustment for the child's height or weight) (Table 4*A*, Table S2). Fat mass (FM), BMI, and grip strength were similar between the two groups (Table 3*A*).

When stratified by sex (Table 4*B*), associations remained in the same direction for both boys and girls, but were not statistically significant. The strength of the associations between cholecalciferol supplementation and bone and lean mass outcomes appeared stronger in girls, for example in the case of WBLH aBMD (boys: β = 0.13 [95% CI, −0.10 to 0.37] SD, *p* = 0.26; girls: β = 0.22 [95% CI, −0.04 to 0.48] SD, *p* = 0.10) and lean mass (boys: β = 0.09 [95% CI, −0.13 to 0.31] SD, *p* = 0.42; girls: β = 0.21 [95% CI, −0.03 to 0.46] SD, *p* = 0.09).

### Interactions between randomization group, season of birth, baseline maternal 25(OH)D, child's calcium intake from milk, or child's physical activity

**Fig. 3:**
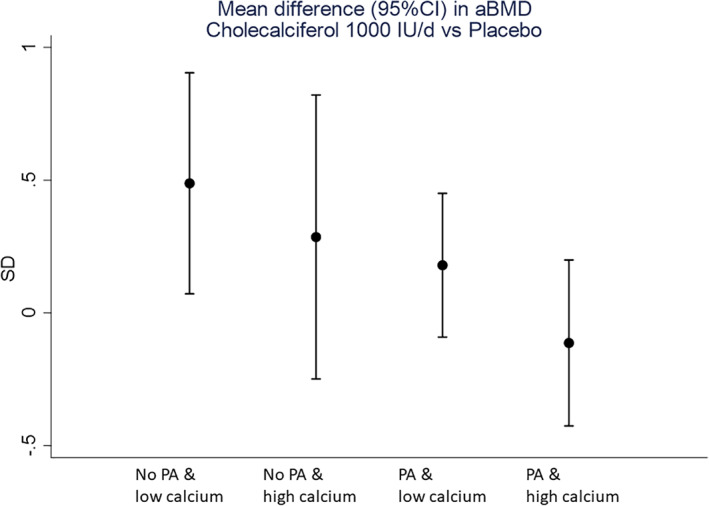
Mean difference in WBLH aBMD by maternal randomization group, stratified by childhood calcium intake (milk consumption below or above 0.5 pints per day) and physical activity (participation or not in organized physical activity).

In the hypothesis‐based interaction analysis, we observed evidence of interactions between the mother's randomization group and the child's calcium intake from milk and on bone outcomes (BA, BMC, aBMD, but not scBMC) at 4 years of age. There was also evidence of an interaction between the child's participation in organized physical activity and aBMD (Table 5*A*,*B*), but there was no evidence of treatment interactions with season of birth (when divided as either two or four seasons) or maternal baseline 25(OH)D (Table S3). There was evidence of a synergistic effect by calcium intake from milk and physical activity status, with the mean difference in aBMD (0.49 [95% CI, 0.07 to 0.90] SD, *p* = 0.022) by maternal randomization group in the children who had low calcium intake from milk and undertook no organized physical activity (Fig. 3; Table S4).

**Table 5:** Associations Between Maternal Treatment Group (Cholecalciferol 1000 IU/d Versus Placebo) and Whole Body Less Head Bone Outcomes in Their Children Assessed at Age 4 Years, Adjusted for Child's Age and Sex (*A*) Stratified by 4‐Year Median Calcium Intake (Estimated as 341 mg Calcium Per Day)

(*A*)	Up to 341 mg Ca/d				More than 341 mg Ca/d				
WBLH DXA outcomes	*n*	β (SD)	95% CI	*p*	*n*	β (SD)	95% CI	*p*	*p* interaction
BA	281	0.16	−0.07, 0.38	0.17	208	−0.20	−0.48, 0.08	0.16	0.006
BMC	281	0.27	0.04, 0.50	0.02	208	−0.11	−0.38, 0.17	0.44	0.004
aBMD	281	0.30	0.07, 0.53	0.01	208	−0.01	−0.28, 0.26	0.94	0.02
scBMC	279	0.08	−0.16, 0.31	0.51	207	0.19	−0.08, 0.46	0.18	0.97

(*B*)	No organized physical activity				Organized physical activity				
WBLH DXA outcomes	*n*	β (SD)		*p*	*n*	β (SD)	95% CI	*p*	*p* interaction
BA	162	0.09	−0.23, 0.41	0.58	327	−0.00	−0.21, 0.20	0.98	0.54
BMC	162	0.30	−0.03, 0.62	0.07	327	0.05	−0.16, 0.26	0.66	0.16
aBMD	162	0.42	0.10, 0.75	0.01	327	0.06	−0.14, 0.26	0.57	0.04
scBMC	162	0.29	−0.01, 0.59	0.06	324	0.04	−0.18, 0.26	0.73	0.19

(*A*) Interaction *p* values between maternal treatment group and child calcium intake from milk are shown. (*B*) Stratified by 4‐year participation in organized physical activity. Interaction *p* values between maternal treatment group and child physical activity are shown.

scBMC = size‐corrected BMC (bone mineral content for bone area, height, and weight).

## Discussion

Maternal cholecalciferol supplementation in pregnancy of 1000 IU daily from 14 weeks of gestation to delivery led to greater aBMD and a trend toward greater BMC in their children at 4 years of age, with evidence of a larger effect in the context of lower childhood calcium intake from milk and physical activity. Furthermore, there appeared to be a beneficial effect of the maternal intervention on offspring lean mass, but no effect on fat mass.

### Comparison with other intervention studies

Other than the MAVIDOS trial, only a few very small intervention studies, until recently, have investigated the effects of antenatal vitamin D supplementation on offspring bone mineralization.^(^ ^10^ ^)^ In these studies, the number of offspring with bone assessments ranged from 25 to 64 individuals, assessed using single‐photon absorptiometry rather than DXA in the earliest trial, and with marked differences in population (UK Asians^(^ ^23^ ^)^ Iran,^(^ ^24^ ^)^ or India^(^ ^25^ ^)^), dose (100 IU/d up to 60,000 IU every 4 weeks) and trial design (randomized/nonrandomized, blinded/nonblinded). The conclusions that can be drawn from the results of these small trials are therefore limited. Recently, findings from a Danish randomized placebo‐controlled trial set within the Copenhagen Prospective Studies on Asthma in Childhood (COPSAC_2010_) demonstrated comparable results to ours and have also demonstrated a beneficial effect of maternal vitamin D supplementation in reducing childhood fractures.^(^ ^26^, ^27^ ^)^ The trials differed in design, both in terms of entry criteria (COPSAC: no 25(OH)D criteria; MAVIDOS: screening 25(OH)D between 25 and 100 nmol/L), dose (COPSAC: 2800 IU/d cholecalciferol versus 400 IU/d; MAVIDOS: 1000 IU/d vs placebo) and timing of intervention (COPSAC: 24 weeks of gestation until 1 week after delivery; MAVIDOS: 14 weeks of gestation until delivery).

In COPSAC_2010_, the differences in WBLH BMC and aBMD at 6 years of age were equivalent to 0.15 and 0.2 SD, respectively, and thus of comparable magnitude to the differences observed in MAVIDOS. The authors adjusted bone relationships for weight and lean for height; it is important that great care is taken in the interpretation of body size‐adjusted bone measures, because there is substantial collinearity between DXA skeletal measures, height, and weight. In part, this is due to height being one dimension of bone area (BA), and thus the envelope within which BMC is contained. Additionally, DXA aBMD is systematically positively biased by greater body size, as a result of the DXA methodology.^(^ ^28^ ^)^ Furthermore, greater skeletal size (leading to greater measured aBMD) necessitates greater lean and fat mass to sustain it. Likewise, greater fat mass needs greater lean mass to enable locomotion.^(^ ^29^ ^)^ Such considerations are very important in growing children, particularly given the expected changes in relative body composition through the end of infancy into later childhood (adiposity rebound) and may mean, for example, that associations at 6 years may not be apparent at 4 years.^(^ ^30^, ^31^ ^)^ Thus, we took a sequential approach to size correction, starting with BA as the overall skeletal size and BMC as the overall mineral content. aBMD gives part size correction and scBMC a fully size‐corrected measure. We additionally investigated whether differences in BA, BMC, or aBMD might be mediated through current height or weight, finding evidence of attenuation in the relationships. Taken together, our findings suggest that body size contributes to, but does not completely explain, the bone differences observed. Indeed, because aBMD was the most strongly affected by maternal cholecalciferol supplementation, this may reflect the disproportionate effect of maternal vitamin D on mineralization within the skeletal envelope, rather than greatly increased envelope size (which would lead to greater effects on BMC and bone area). A trend toward an association between maternal cholecalciferol supplementation and lean mass was also seen, and because lean mass is important for skeletal mineralization this may also have been contributing to the bone associations. Such an effect on lean mass could be mediated through a direct effect of vitamin D acting through the vitamin D receptor in skeletal muscle^(^ ^32^ ^)^ or through epigenetic modification of genes determining skeletal muscle and/or overall body size.^(^ ^13^ ^)^ Indeed, we have previously demonstrated differences in methylation of the *RXRA* gene by maternal randomization to cholecalciferol in this study.

### Interaction with calcium intake from milk and physical activity

Our finding of interactions between maternal randomization group and the child's calcium intake from milk and physical activity on bone outcomes is intriguing. There is good evidence that both calcium and vitamin D are threshold nutrients; ie, levels above a threshold are not additionally beneficial.^(^ ^33^ ^)^ 1,25(OH)_2_‐vitamin D (the active form to which 25(OH)D, the circulating storage form, is converted) acts on the small intestine to increase fractional calcium absorption. This is likely to be more necessary in states of low calcium intake; indeed, there is evidence that the biochemical consequences of vitamin D deficiency are more marked when there is concomitant low dietary calcium intake.^(^ ^34^ ^)^ In the present case, we are considering the child's calcium intake from milk in relation to their in utero vitamin D exposure as a result of maternal randomization to cholecalciferol or placebo. Consistent with these findings, we have previously demonstrated, in a population with adequate vitamin D levels, that lower calcium intake during pregnancy is associated with lower bone mass in childhood.^(^ ^35^ ^)^ One possibility is that the low calcium intake from milk of the child reflects an inherited environment of habitual low calcium intake, and thus low calcium intake of the mother during pregnancy. This might lead to greater scope for the vitamin D supplementation to benefit the neonatal skeleton in utero, tracking through to 4 years of age. Alternatively, if the maternal vitamin D supplementation altered the setpoint for vitamin D metabolism in the offspring, then again there would be more scope for this alteration to improve bone accrual in those below compared with those above a particular level of calcium intake during childhood. Consistent with such a notion, we have demonstrated that gestational vitamin D supplementation leads to altered perinatal offspring epigenetic marking in the *RXRA* gene,^(^ ^13^ ^)^ a key part of vitamin D signaling. Interaction between calcium intake and physical activity on bone mineral accrual in children has been demonstrated,^(^ ^21^, ^36^ ^)^ as well as potential effects of early vitamin D exposure on bone mechanobiology, both in animal models^(^ ^37^ ^)^ and in a small subset of MAVIDOS children.^(^ ^38^ ^)^ Similar considerations thus apply to the interaction with childhood physical activity. Together these findings suggest that this maternal intervention is likely to be of most benefit where low maternal vitamin D status in pregnancy is followed by poor calcium nutrition and low levels of physical activity in the offspring.^(^ ^13^ ^)^

### Public health implications

The longer‐term impact of our findings remains to be demonstrated, and indeed full follow‐up of the MAVIDOS children at 6–8 years of age across all three study centers is ongoing. Our results provide further evidence that maternal pregnancy vitamin D supplementation, here administered using an approach completely congruent with UK obstetric care pathways, does influence offspring skeletal development in a way that is likely to have relevance for future bone health. Although the impact on neonatal bone mass was only observed for births that occurred during winter months, here we documented greater bone mass at 4 years unstratified by season. The difference in neonatal BMC was around 0.1 SD in the direction of benefit from vitamin D supplementation, but did not meet the prespecified threshold for statistical significance,^(^ ^12^ ^)^ whereas at 4 years of age we see an effect of slightly greater magnitude supported by greater statistical evidence. That the magnitude, or even direction, of early life effects may change with increasing offspring age has been previously demonstrated in regard to gestational 25(OH)D and offspring fat mass^(^ ^30^ ^)^: in the Southampton Women's Survey, positive associations were observed between maternal pregnancy 25(OH)D and offspring fat mass at birth, but no association at 4 years and there was an inverse association at 6 years of age. Interestingly, we see a similar pattern for fat mass in MAVIDOS (in so far as we have neonatal and 4‐year assessments to date).^(^ ^12^ ^)^ In adults, a 0.5 SD reduction in aBMD is associated with an approximate doubling in fracture risk.^(^ ^39^ ^)^ The 0.17 SD improvement in aBMD associated with maternal gestational vitamin D supplementation observed in this study would therefore be consistent with the notion that this gestational intervention might, if adequately sustained into adult life, lead to a reduction in the risk of fractures in older age.^(^ ^40^ ^)^

### Strengths and limitations

We present the preplanned 4‐year assessment of children born to the largest primarily bone outcome‐focused trial of maternal pregnancy vitamin D supplementation to date, using the gold standard measure of bone and body composition.^(^ ^12^ ^)^ However, there are some limitations that must be considered. First, we could not, as a result of stipulations made during the ethics approval process, include participants with 25(OH)D concentrations <25 nmol/L at screening for trial enrolment. In addition, our study population did not include many members of ethnic minorities. Both of these points are likely to lead to a conservative bias, reducing any differences observed rather than the opposite, but may affect the generalizability of our findings. Second, DXA assessment in children presents some difficulties, because children are prone to move and have low absolute BMC. Appropriate pediatric software was used and the validity of the technique in small animals has been documented.^(^ ^41^ ^)^ Third, although we could not exclude the possibility that some participants were taking vitamin D in addition to the study medication and we did not have measures of serum 25(OH)D in the children, supplement use was recorded and did not differ between the groups. Fourth, we had limited ability to control for detailed dietary, physical activity, and environmental factors (such as ambient ultraviolet B [UVB] exposure) for the children at 4 years, but there is no reason to suppose that such exposures would have systematically differed by maternal randomization group. Finally, although the 4‐year follow‐up was specified in the original protocol, it does of course not represent a primary analysis and was carried out at the Southampton site only, due to funding constraints. However, the Southampton site did represent the majority of recruitment in the main trial. These findings will require replication in other studies, which indeed is planned in a further trial in Southampton, UK.^(^ ^42^ ^)^

## Conclusions

In conclusion, our results, from this secondary analysis of the MAVIDOS randomized controlled trial, are consistent with the notion that maternal pregnancy vitamin D supplementation might have a persisting influence on offspring skeletal development. If the effect of antenatal cholecalciferol supplementation on BMD were to be sustained throughout childhood and puberty to peak bone mass, it would be expected to reduce the future burden of adult fractures. Additionally, our findings suggest that such effects might be obtained at modest doses (1000 IU/d) administered over a time course consistent with typical antenatal care pathways.

## Author Contributions

**Elizabeth M. Curtis:** Conceptualization; formal analysis; investigation; methodology; project administration; writing – original draft. **Rebecca J. Moon:** Conceptualization; formal analysis; investigation; methodology; project administration; writing – original draft. **Stefania D'Angelo:** Data curation; formal analysis; writing – original draft. **Sarah R. Crozier:** Conceptualization; data curation; formal analysis; methodology; writing – review and editing. **Nicholas J. Bishop:** Conceptualization; investigation; methodology; writing – review and editing. **Jaya Sujatha Gopal‐Kothandapani:** Investigation; methodology; project administration; writing – review and editing. **Stephen H. Kennedy:** Conceptualization; investigation; methodology; writing – review and editing. **Aris T. Papageorghiou:** Conceptualization; investigation; methodology; writing – review and editing. **Robert Fraser:** Conceptualization; investigation; methodology; writing – review and editing. **Saurabh V. Gandhi:** Data curation; methodology; project administration; writing – review and editing. **Inez Schoenmakers:** Investigation; methodology; validation; writing – review and editing. **Ann Prentice:** Conceptualization; methodology; validation; writing – review and editing. **Hazel M. Inskip:** Conceptualization; data curation; funding acquisition; investigation; methodology; supervision; writing – review and editing. **Keith M. Godfrey:** Conceptualization; funding acquisition; investigation; methodology; supervision; writing – review and editing. **M. Kassim Javaid:** Conceptualization; funding acquisition; investigation; methodology; writing – review and editing. **Richard Eastell:** Conceptualization; investigation; methodology; validation; writing – review and editing. **Cyrus Cooper:** Conceptualization; funding acquisition; investigation; methodology; supervision; writing – original draft; writing – review and editing. **Nicholas C. Harvey:** Conceptualization; formal analysis; funding acquisition; investigation; methodology; project administration; supervision; writing – original draft; writing – review and editing.

## Conflicts of Interests

EMC reports honoraria/travel support from Eli Lilly, Pfizer, and UCB outside the submitted work. NJB reports remuneration from Internis Pharmaceuticals Ltd, outside the submitted work. ATP reports grants from Versus Arthritis, Medical Research Council, National Institute for Health Research, Bupa Foundation, BBSRC, and EU outside the submitted work. KMG reports reimbursement for speaking at Nestle Nutrition Institute conferences, grants from Abbott Nutrition & Nestec, outside the submitted work; in addition, KMG has a patent Phenotype Prediction pending, a patent Predictive Use of CpG Methylation pending, and a patent Maternal Nutrition Composition pending, not directly related to this work. MKJ reports personal fees from Stirling Anglia, Consilient Health and Internis, outside the submitted work. RE reports grants from Amgen, grants and personal fees from IDS, grants from Alexion, grants and personal fees from Roche, personal fees from GSK Nutrition, personal fees from Mereo, personal fees from Sandoz, grants and personal fees from Nittobo, personal fees from AbbVie, personal fees from Samsung, personal fees from Haoma Medica, personal fees from Elsevier, personal fees from CL Bio, personal fees from FNIH, personal fees from Viking, personal fees from UCSF, personal fees from Biocon, from Lyramid, outside the submitted work. CC reports personal fees from ABBH, Amgen, Eli Lilly, GSK, Medtronic, Merck, Novartis, Pfizer, Roche, Servier and Takeda, outside the submitted work. NCH reports personal fees, consultancy, lecture fees and honoraria from Alliance for Better Bone Health, AMGEN, MSD, Eli Lilly, Servier, Shire, UCB, Consilient Healthcare, Kyowa Kirin and Internis Pharma, outside the submitted work. RJM, SD, SRC, JSGK, SHK, RF, SVG, IS, AP, and HMI have nothing to disclose. Of the MAVIDOS group: N. Arden has received honoraria, held advisory board positions (which involved receipt of fees), and received consortium research grants, respectively, from: Merck, grants from Roche, personal fees from Smith & Nephew, Nicox, Flexion, grants from Bioiberica, Novartis, and personal fees from Bioventus and Freshfields, outside the submitted work. M.Z. Mughal has received lecture fees from Abbott Nutrition & Thornton & Ross, outside the submitted work. A. Carr, M. Clynes, E. Dennison, D. Reid, and S. Woolford have nothing to disclose.

## Data Sharing

Please contact the Chief Investigator, Cyrus Cooper (cc@mrc.soton.ac.uk) with data sharing requests.

### Peer Review

The peer review history for this article is available at https://publons.com/publon/10.1002/jbm4.10651.

## Supporting information


**Table S1** Comparison of the mothers attending the 4 year follow up visit with mothers remaining in the study until delivery, groups combined, and by randomization group.
**Table S2** Associations between maternal treatment group (cholecalciferol 1000 IU/d versus placebo) and whole body less head DXA/body composition outcomes in their children assessed at age 4 years.
**Table S3** Interaction between maternal treatment group (cholecalciferol 1000 IU/d versus placebo) and (i) child calcium intake from milk; (ii) organized physical activity; (iii) maternal baseline pregnancy 25(OH)D; and (iv) season of offspring delivery on whole body less head DXA/body composition outcomes assessed at age 4 years.
**Table S4** Mean difference in child WBLH aBMD at 4 years by maternal treatment group (cholecalciferol 1000 IU/dy versus placebo), stratified by childhood calcium intake and physical activity.
