# Supplementary material for: Pregnancy Vitamin D Supplementation and Childhood Bone Mass at Age 4 Years: Findings From the Maternal Vitamin D Osteoporosis Study (MAVIDOS) Randomized Controlled Trial
Source: JBMR Plus. 2022 Jun 11;6(7):e10651. doi: 10.1002/jbm4.10651 (PMC9289979; doi:10.1002/jbm4.10651)
Supplement: Supplementary file 1 — Table S1 Comparison of the mothers attending the 4 year follow up visit with mothers remaining in the study until delivery, groups combined, and by randomization group. Table S2 Associations between maternal treatment group (cholecalciferol 1000 IU/d versus placebo) and whole body less head DXA/body composition outcomes in their children assessed at age 4 years. Table S3 Interaction between maternal treatment group (cholecalciferol 1000 IU/d versus placebo) and (i) child calcium intake from milk; (ii) organized physical activity; (iii) maternal baseline pregnancy 25(OH)D; and (iv) season of offspring delivery on whole body less head DXA/body composition outcomes assessed at age 4 years. Table S4 Mean difference in child WBLH aBMD at 4 years by maternal treatment group (cholecalciferol 1000 IU/dy versus placebo), stratified by childhood calcium intake and physical activity. [file JBM4-6-e10651-s001.docx]

**Supplemental Table 1:** Comparison of the mothers attending the 4 year follow up visit with mothers remaining in the study until delivery, groups combined, and by randomisation group.

| **Maternal measures** | **n** | **4-year visit attendees** | **n** | **4-year visit non-attendees** | **p difference** |
| --- | --- | --- | --- | --- | --- |
| Maternal age at delivery (years) | 564 | 32.0 (4.7) | 402 | 29.9 (5.5) | < 0.001 |
| Ethnicity (white vs non-white)^*^ | 532 | 508 (95.5) | 397 | 373 (94.0) | 0.30 |
| Parity (nulliparous)^*^ | 532 | 229 (43.1) | 395 | 174 (44.1) | 0.76 |
| Educational attainment (A level or higher)^*^ | 530 | 437 (82.5) | 396 | 281 (71.0) | < 0.001 |
| Height (cm) | 531 | 166.0 (6.4) | 396 | 165.2 (6.5) | 0.07 |
| BMI (kg/m^2^)^+^ | 531 | 25.0 (22.6, 29.2) | 396 | 25.1 (22.5, 29.3) | 0.75 |
| Early pregnancy smoking^*^ | 533 | 25 (4.7) | 397 | 47 (11.8) | < 0.001 |
| Late pregnancy smoking^*^ | 499 | 25 (5.0) | 341 | 33 (9.7) | 0.009 |
| Moderate/strenuous physical activity in LP (hrs/week) | 355 | 0.9 (0.6) | 193 | 0.9 (0.6) | 0.36 |
| Offspring sex (male)^*^ | 563 | 305 (54.2) | 402 | 212 (52.7) | 0.66 |

| **PLACEBO** | | | | | |
| --- | --- | --- | --- | --- | --- |
| **Maternal measures** | **n** | **4-year visit attendees** | **n** | **4-year visit non-attendees** | **p difference** |
| Maternal age at delivery (years) | 286 | 32.1 (4.7) | 201 | 29.8 (5.5) | <0.001 |
| Ethnicity (white vs non-white)^*^ | 269 | 260 (96.7) | 198 | 180 (90.9) | 0.009 |
| Parity (nulliparous)^*^ | 267 | 114 (42.7) | 197 | 91 (46.2) | 0.45 |
| Educational attainment (A level or higher)^*^ | 266 | 216 (81.2) | 197 | 140 (71.1) | 0.01 |
| Height (cm) | 265 | 166.3 (6.4) | 198 | 165.0 (6.9) | 0.03 |
| BMI (kg/m^2^)^+^ | 265 | 25.5 (22.8,29.6) | 198 | 25.5 (22.8,30.0) | 0.93 |
| Early pregnancy smoking^*^ | 268 | 14 (5.2) | 198 | 21 (10.6) | 0.03 |
| Late pregnancy smoking^*^ | 254 | 13 (5.1) | 175 | 15 (8.6) | 0.16 |
| Moderate/strenuous physical activity in LP (hrs/week) | 181 | 0.8 (0.5) | 98 | 0.9 (0.5) | 0.25 |
| Offspring sex (male)^*^ | 285 | 144 (50.5) | 201 | 109 (54.2) | 0.42 |
|  |  |  |  |  |  |
| **CHOLECALCIFEROL** | | | | | |
| **Maternal measures** | **n** | **4-year visit attendees** | **n** | **4-year visit non-attendees** | **p difference** |
| Maternal age at delivery (years) | 278 | 32.0 (4.7) | 201 | 29.9 (5.5) | <0.001 |
| Ethnicity (white vs non-white)^*^ | 263 | 248 (94.3) | 199 | 193 (97.0) | 0.17 |
| Parity (nulliparous)^*^ | 265 | 115 (43.4) | 198 | 83 (41.9) | 0.75 |
| Educational attainment (A level or higher)^*^ | 264 | 221 (83.7) | 199 | 141 (70.9) | 0.001 |
| Height (cm) | 266 | 165.6 (6.3) | 198 | 165.4 (6.2) | 0.70 |
| BMI (kg/m^2^)^+^ | 266 | 24.9 (22.3,28.5) | 198 | 24.6 (22.2,28.7) | 0.62 |
| Early pregnancy smoking^*^ | 265 | 11 (4.2) | 199 | 26 (13.1) | <0.001 |
| Late pregnancy smoking^*^ | 245 | 12 (4.9) | 166 | 18 (10.8) | 0.02 |
| Moderate/strenuous physical activity in LP (hrs/week) | 174 | 0.9 (0.7) | 95 | 0.9 (0.6) | 0.78 |
| Offspring sex (male)^*^ | 278 | 161 (57.9) | 201 | 103 (51.2) | 0.15 |

Shown as mean (SD), n (%)* or median (IQR)+

**Supplemental Table 2:** Associations between maternal treatment group (cholecalciferol 1000 IU/ day versus placebo) and whole body less head DXA/ body composition outcomes in their children assessed at age 4 years.

| **WBLH DXA outcomes** | **Cholecalciferol vs placebo** | | | | | | | | | | | |
| --- | --- | --- | --- | --- | --- | --- | --- | --- | --- | --- | --- | --- |
|  | **Model 1: adjusted for age, sex** | | | | **Model 2: adjusted for age, sex, child height** | | | | **Model 3: adjusted for age, sex, child weight** | | | |
|  | **N** | **β (SD)** | **95% CI** | **P value** | **N** | **β (SD)** | **95% CI** | **P value** | **N** | **β (SD)** | **95% CI** | **P value** |
| BA | 489 | 0.01 | -0.16,0.19 | 0.87 | 488 | -0.08 | -0.22,0.05 | 0.24 | 489 | -0.06 | -0.20,0.09 | 0.44 |
| BMC | 489 | 0.12 | -0.06,0.30 | 0.18 | 488 | 0.01 | -0.11,0.13 | 0.89 | 489 | 0.03 | -0.08,0.14 | 0.63 |
| aBMD | 489 | 0.17 | 0.00,0.35 | 0.05 | 488 | 0.08 | -0.06,0.21 | 0.26 | 489 | 0.09 | -0.03,0.20 | 0.14 |
| scBMC* | 486 | 0.12 | -0.05,0.30 | 0.17 | - | - | - | - | - | - | - | - |
| Lean | 491 | 0.15 | -0.02,0.31 | 0.08 | 490 | 0.04 | -0.07,0.15 | 0.43 | 491 | 0.06 | -0.03,0.16 | 0.21 |
| Fat | 491 | -0.01 | -0.18,0.16 | 0.91 | 490 | -0.07 | -0.22,0.08 | 0.36 | 491 | -0.09 | -0.20,0.02 | 0.10 |

*scBMC=size-corrected BMC (BMC for BA, height and weight)

**Supplemental Table 3:** Interaction between maternal treatment group (cholecalciferol 1000 IU/ day versus placebo) and 1) child calcium intake from milk; 2) organised physical activity; 3) maternal baseline pregnancy 25(OH)D; and 4) season of offspring delivery on whole body less head DXA/ body composition outcomes assessed at age 4 years.

|  |  |  |  |  |  |  |
| --- | --- | --- | --- | --- | --- | --- |
| **WBLH DXA outcomes** | **child calcium intake from milk (adjusted for age, sex)** | | **child’s physical activity (yes/no) (adjusted for age, sex)** | **baseline 25(OH)D (adjusted for age, sex)** | **Season of birth (2 seasons) (adjusted for age, sex)** | **Season of birth (4 seasons) (adjusted for age, sex)** |
| BA | 0.006 | | 0.54 | 0.74 | 0.17 | 0.17 |
| BMC | 0.004 | | 0.16 | 0.44 | 0.25 | 0.48 |
| aBMD | 0.02 | | 0.04 | 0.31 | 0.66 | 0.76 |
| scBMC* | 0.97 | | 0.19 | 0.28 | 0.39 | 0.82 |
| Lean | 0.02 | | 0.29 | 0.61 | 0.48 | 0.61 |
| Fat | 0.005 | | 0.15 | 0.71 | 0.02 | 0.12 |

*scBMC=size-corrected BMC (BMC for BA, height and weight)

**Supplemental Table 4:** Mean difference in child WBLH aBMD at 4 years by maternal treatment group (cholecalciferol 1000 IU/ day versus placebo), stratified by childhood calcium intake and physical activity.

| **Group** | **β (95%CI)** | **p difference** |
| --- | --- | --- |
| No PA & low calcium | 0.49 (0.07,0.90) | 0.02 |
| No PA & high calcium | 0.29 (-0.25,0.82) | 0.29 |
| PA & low calcium | 0.18 (-0.09,0.45) | 0.19 |
| PA & high calcium | -0.11 (-0.43,0.20) | 0.48 |

P value for a 3-way interaction between PA, calcium intake and maternal treatment group = 0.81

Low/high calcium = milk intake at 4 years below or above 341mg Calcium per day; PA = participates in organised physical activity; no PA = no participation in organised physical activity
